# The Transcription Factor IRF9 Promotes Colorectal Cancer via Modulating the IL-6/STAT3 Signaling Axis

**DOI:** 10.3390/cancers14040919

**Published:** 2022-02-12

**Authors:** Bhesh Raj Sharma, Rajendra Karki, Balamurugan Sundaram, Yaqiu Wang, Peter Vogel, Thirumala-Devi Kanneganti

**Affiliations:** 1 Department of Immunology, St. Jude Children’s Research Hospital, Memphis, TN 38105, USA; bhesh.sharma@stjude.org (B.R.S.); rajendra.karki@stjude.org (R.K.); balamurugan.sundaram@stjude.org (B.S.); yaqiu.wang@stjude.org (Y.W.); 2 Animal Resources Center and the Veterinary Pathology Core, St. Jude Children’s Research Hospital, Memphis, TN 38105, USA; peter.vogel@stjude.org

**Keywords:** interferons, IRF9, colitis, colorectal cancer, tumorigenesis, IL-6, STAT3, AOM, DSS, cell death

## Abstract

**Simple Summary:**

Colorectal cancer (CRC) is the second most common cause of cancer-related death worldwide. While the exact causes and prognosis of CRC are complex, colonic inflammation is the major predisposing factor for the development of CRC. The aim of our study was to investigate the physiological function of interferon regulatory factor 9 (IRF9) in CRC. We found that mice deficient in IRF9 developed fewer tumors compared with their corresponding WT littermates in mouse models of CRC. Mechanistically, IRF9 was required for IL-6 production which drove the activation of STAT3, leading to the development of tumors in the colon. Overall, these findings will be important to inform the development of therapeutic strategies to improve outcomes for patients with this deadly cancer and other diseases where the IRF9-mediated production of IL-6 can be therapeutically modulated.

**Abstract:**

Colorectal cancer (CRC) is a leading cause of cancer-related deaths worldwide, and innate immune responses and inflammation are known to affect the course of disease. Interferon (IFN) signaling in particular is critical for modulating inflammation-associated diseases including CRC. While the effects of IFN signaling in CRC have been studied, results have been conflicting. Furthermore, individual molecules in the IFN pathway that could be therapeutically targeted have distinct functions, with many of their diverse roles in CRC remaining unclear. Here, we found that IRF9 had an oncogenic effect in CRC; loss of IRF9 reduced tumorigenesis in both azoxymethane (AOM)/dextran sodium sulfate (DSS)-induced and spontaneous CRC models. IRF9 also reduced DSS-induced colitis and inflammation in the colon, but it had no effect on the NF-κB and MAPK signaling activation. Instead, IRF9 enhanced the transcription and production of the inflammatory cytokine IL-6. By promoting IL-6 release, IRF9 drove the activation of pro-oncogenic STAT3 signaling in the colon. Overall, our study found that IRF9 promoted the development of CRC via modulation of the IL-6/STAT3 signaling axis, identifying multiple potential targets and suggesting new therapeutic strategies for the treatment of CRC.

## 1. Introduction

Colorectal cancer (CRC) is the second most common cause of cancer-related death worldwide. More than 1.9 million new CRC cases and 935,000 deaths were estimated to occur in 2020 [1]. While the exact causes and prognosis of CRC are complex, colonic inflammation is considered to be the major predisposing factor for the development of CRC, suggesting that innate immune responses and inflammation are key determinants in the progression of CRC [2].

During infectious and non-infectious inflammatory diseases, including CRC, the interferon (IFN) cytokine family is important for maintaining tissue homeostasis and initiating immune responses [3]. IFN production and IFN signaling are controlled by IFN regulatory factors (IRFs), a group of transcription factors which modulate cellular inflammation in response to innate and adaptive immune sensing [4]. When type I IFNs are produced and released, they bind their cognate receptors, leading to the activation of signal transducer and activator of transcription 1 (STAT1). STAT1 then dimerizes and complexes with STAT2 and IRF9 to form IFN-stimulated gene (ISG) factor 3 (ISGF3), which translocates to the nucleus and regulates the transcription of a large number of cytokines and chemokines to modulate host immune responses [5].

While IFN signaling provides host protection in acute viral infections, such as influenza infection, it can have either protective or deleterious effects in bacterial infections depending upon the infecting pathogen [6]. For instance, IFN signaling is protective in mice infected with *Streptococcus pyogenes* [7] and *Helicobacter pylori* [8], but it is detrimental in the case of *Francisella novicida* [9], *Listeria monoocytogenes* [10], and *Salmonella* Typhimurium [11] infections. In addition, IFN signaling has been reported to drive context-specific immune responses in inflammation-mediated diseases [12]. For example, while proper IFN signaling is crucial to regress tumor formation [13], aberrant IFN signaling is detrimental in systematic lupus erythematosus [14] and in cytokine storm-associated diseases such as sepsis, hemophagocytic lymphohistiocytosis, and SARS-CoV-2 infection [15,16].

In many cases, inflammation exacerbates the development of tumors. In particular, colonic inflammation can increase mutagenesis and predispose the colon to tumor formation [17,18]. Although the role of IFN signaling in CRC has been studied by several groups, their results are confounding. In the azoxymethane (AOM)/dextran sodium sulfate (DSS)-induced mouse model of CRC, IRF3-deficient mice develop more tumors [19], while type I IFN receptor (IFNAR)-deficient mice develop a similar number of tumors when compared with WT mice [20]. Additionally, we recently reported the tumor suppressive function of IRF1 during CRC, as IRF1 potentiates inflammatory cell death, PANoptosis, in cancer cells [21,22]. STAT1 and STAT2, two of the key components of the ISGF3 complex that regulates the production of many ISGs, are also reported to have distinctive functions in CRC [23,24,25]. However, the function of the third member of the ISGF3 complex, IRF9, in CRC is currently unknown. Given the conflicting roles of IFN signaling reported in CRC to date, it is important to understand the function of these molecules to identify targets for therapeutic intervention.

Therefore, here we investigated the physiological function of IRF9 in CRC. We found that mice deficient in IRF9 developed fewer tumors compared with their corresponding WT littermates in the mouse model of AOM/DSS-induced colitis-associated CRC. Additionally, deficiency of IRF9 reduced tumor burden in a spontaneous mouse model of CRC. Mechanistically, we found that while IRF9 was not required for the activation of NF-κB and p38 mitogen activated protein kinase (MAPK), it was required for IL-6 production and the subsequent STAT3 activation, leading to the development of tumors in the colon. Overall, our study identifies a previously unknown role for IRF9 in controlling IL-6 production to drive STAT3-mediated tumorigenesis in CRC. These findings will be important to inform the development of therapeutic strategies to improve outcomes for patients with this deadly cancer and other diseases where the IRF9-mediated production of IL-6 plays a pathological role.

## 2. Materials and Methods

### 2.1. Mice

*Irf9*^−/−^ mice [26] have been previously described. C57BL/6J-*Apc*^Min/+^/J mice (002020, The Jackson Laboratory) were used as an *Apc*^Min^ model of colorectal tumorigenesis. All mice were bred at St. Jude Children’s Research Hospital. Animal studies were conducted under protocols approved by St. Jude Children’s Research Hospital’s committee on the use and care of animals.

### 2.2. AOM/DSS Model of Colorectal Tumorigenesis

Both male and female littermate mice were injected with 10 mg of AOM (Sigma, St. Louis, MO, USA) per kg body weight according to previously established protocols [27]. Five days later, 2.5% DSS (Affymetrix, Santa Clara, CA, USA, 9011-18-1) was given in the drinking water for 6 days followed by regular drinking water for 2 weeks. This cycle was repeated twice with 2% DSS, and mice were sacrificed on day 80. For day 14 samples, mice were injected with AOM and, after 5 days, 2.75% DSS was administered for 6 days. Mice were then administered regular drinking water for 3 days and sacrificed.

### 2.3. Apc^Min^ Model of Colorectal Tumorigenesis

The *Apc*^Min/+^ mice were genetically crossed with *Irf9*^−/−^ mice to generate littermate *Apc*^Min/+^, *Apc*^Min/+^*Irf9*^−/+^, and *Apc*^Min/+^*Irf9*^−/−^ mice. Mice from these littermates were harvested at 70 days old to assess inflammatory markers and 120 days old to assess the prevalence of tumors in the colon.

### 2.4. Histology and Microscopy Analysis

Colons were rolled into a “Swiss roll” and fixed in 10% formalin, then processed and embedded in paraffin by standard techniques. Longitudinal sections of 5 μm thickness were stained with hematoxylin and eosin and examined by a pathologist blinded to the experimental groups. Colitis scores were assigned based on inflammation, ulceration, hyperplasia, and the extent or severity of the damage. Severity scores for inflammation were assigned as follows: 0 = normal (within normal limits); 2 = minimal (mixed inflammation, small, focal, or widely separated, limited to lamina propria); 15 = mild (multifocal mixed inflammation, often extending into submucosa); 40 = moderate (large multifocal lesions within mixed inflammation involving mucosa and submucosa); 80 = marked (extensive mixed inflammation with edema and erosions); 100 = severe (diffuse inflammation with transmural lesions and multiple ulcers). Scores for ulceration were assigned as follows: 0 = normal (none); 2 = minimal (only one small focus of ulceration involving fewer than 5 crypts); 15 = mild (a few small ulcers, up to 5 crypts); 40 = moderate (multifocal ulcers, up to 10 crypts); 80 = marked (multifocal to coalescing ulcers involving more than 10 crypts each); 100 = severe (extensive to diffuse with multiple ulcers covering more than 20 crypts each). Scores for hyperplasia were assigned as follows: 0 = normal; 2 = minimal (some areas with crypts elongated and increased mitoses); 15 = mild (multifocal areas with crypts elongated up to twice the normal thickness, normal goblet cells present); 40 = moderate (extensive areas with crypts up to 2 times normal thickness, reduced goblet cells); 80 = marked (mucosa over twice the normal thickness, hyperchromatic epithelium, reduced or rare goblet cells, possibly foci of arborization); 100 = severe (mucosa twice the normal thickness, marked hyperchromasia, crowding/stacking, absence of goblet cells, high mitotic index and arborization). Damage extent scores were assigned as follows: 0 = normal (rare or inconspicuous lesions); 2 = minimal (less than 5% involvement); 15 = mild (multifocal but conspicuous lesions, 5 to 10% involvement); 40 = moderate (multifocal, prominent lesions, 10 to 50% involvement); 80 = marked (coalescing to extensive lesions or areas of inflammation with some loss of structure, 50 to 90% involvement); 100 = severe (diffuse lesion). pSTAT3 staining was performed according to the manufacturer’s instructions (#9145, CST). Tissues were counterstained with hematoxylin.

### 2.5. Cytokine Measurement by ELISA

Cytokines in the colon or serum were measured by ELISA according to the manufacturer’s instructions. IL-18 was measured using an ELISA kit for IL-18 (Invitrogen, BMS618-3). All other cytokines were measured by a multiplex ELISA (Millipore, Burlington, VT, USA, Cat# MCYTOMAG-70K).

### 2.6. Western Blotting

Proteins were extracted from colon tissue using RIPA lysis buffer supplemented with protease (Roche, St. Louis, MO, USA, 11697498001) and phosphatase inhibitors (Roche, 04906837001) as described previously [28]. For immunoblot analysis of in vitro samples, supernatants were removed, and cells were washed once with PBS, followed by lysis in RIPA buffer and sample loading buffer. Proteins were separated by electrophoresis through 8–12% polyacrylamide gels. Following electrophoretic transfer of proteins onto PVDF membranes (Millipore, IPVH00010), nonspecific binding was blocked by incubation with 5% skim milk, then membranes were incubated with primary antibodies against: p-IκBα (#9241, CST, Danvers, MA, USA), IκBα (#9242, CST), p-p38 (#9211, CST), p38 (#9212, CST), pSTAT3 Tyr705 (#9131, CST), STAT3 (#9139, CST), CASP3 (#9662, CST), cleaved CASP3 (#9661, CST), CASP7 (#9492, CST), cleaved CASP7 (#9491, CST), CASP8 (#AG-20T-0138-C100, Adipogen, San Diego, AK, USA), cleaved CASP8 (#8592, CST), GSDMD (#ab209845, Abcam, Waltham, MA, USA), GSDME (#ab215191, Abcam), IRF9 (#28845, CST), and GAPDH (#5174, CST). Membranes were then washed and incubated with the appropriate horseradish peroxidase (HRP)-conjugated secondary antibodies (Jackson ImmunoResearch Laboratories, anti-rabbit (111–035–047) and anti-mouse (315–035–047)). Proteins were visualized using Luminata Forte Western HRP Substrate (WBLUF0500, Millipore).

### 2.7. Real-Time PCR (RT-PCR) Analysis

RNA was extracted from in vitro or in vivo samples by using TRIzol (Thermo Fisher Scientific, Waltham, MA, USA, cat no. 155960260) or a Mini-Prep Kit (Bio Basic, Amherst, MA, USA, cat. no. BS822322), respectively, in accordance with the manufacturer’s instructions. The isolated RNA was reverse-transcribed into cDNA with a First-Strand cDNA Synthesis Kit (Applied Biosystems, cat. no. 4368814). Real-time quantitative PCR was performed on an ABI 7500 RT-PCR instrument, using 2× SYBR Green (Applied Biosystems, cat. no. 4368706) and the appropriate primers. The sequences for the qRT-PCR primers are listed in Supplementary Table S1.

### 2.8. Cell Culture and Stimulation of Cells

Primary mouse bone marrow-derived macrophages (BMDMs) were cultured as described previously [29]. BMDMs (1 × 10^6^ cells/well) were seeded in 12-well plates. Cells were stimulated with 50 ng/mL LPS (#L2630, Sigma), 10 ng/mL Poly (I:C) (Invivogen, tlrl-pic) or 20 ng/mL IL-6 (Peprotech, Cranbury, NJ, USA, 212-16) for the indicated time for further analysis.

### 2.9. ChIP-Seq Analysis

The pre-processed IRF9 (GSE115435) [30] and H3K4Me3 (GSM1441327) [31] ChIP-Seq data were downloaded from Cistrome database [32] as BED files. The visualization of the genomic region of *Il6* and *Il1b* was performed using Integrated Genome Browser.

### 2.10. Statistical Analysis

Statistical significance was determined by the two-tailed student’s *t*-test or ANOVA using GraphPad Prism v8.0. The specific test used is indicated in the respective figure legends. *p* < 0.05 was considered statistically significant.

## 3. Results

### 3.1. IRF9 Promotes the Development of Colitis-Associated Tumorigenesis

**Figure 1:**
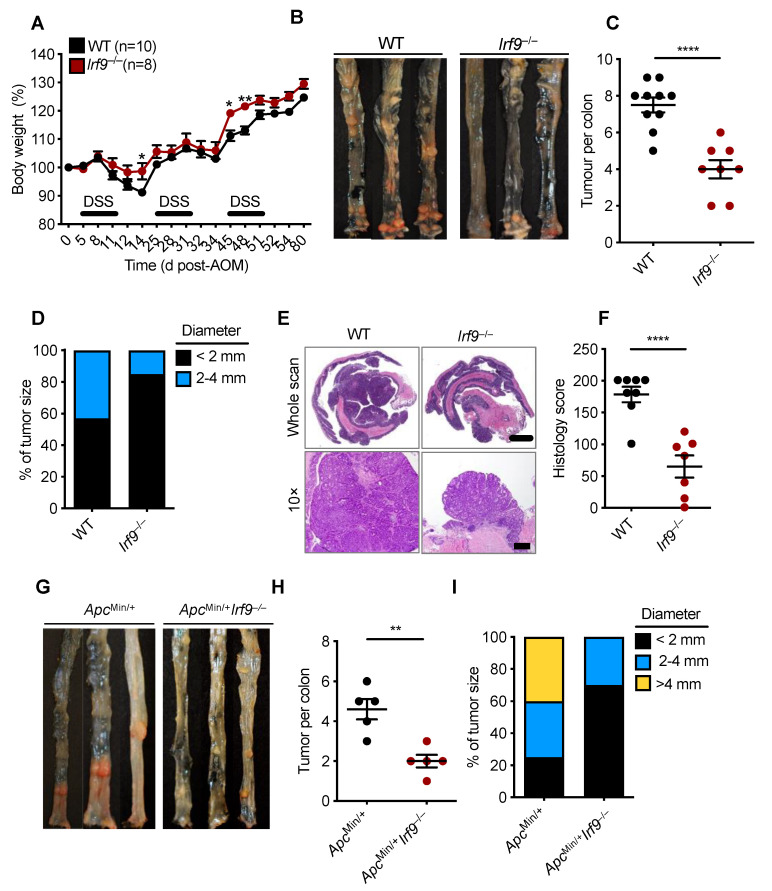
**IRF9-deficient mice are resistant to the development of colorectal cancer**. (**A**) Body weight change of wild type (WT) (n = 10) and *Irf9*^−/−^ (n = 8) littermate mice from one experiment, representative of three independent experiments. (**B**) Representative images of colon tumors in littermate WT and *Irf9*^−/−^ mice 80 days after injection of azoxymethane (AOM). (**C**) Number and (**D**) size of colon tumors in WT (n = 10) and *Irf9*^−/−^ (n = 8) mice. (**E**) Representative hematoxylin and eosin staining of colon tumors; scale bar, 2 mm for whole scan and 500 μm for 10×. (**F**) Histological scores 80 days after injection of AOM in WT (n = 8) and *Irf9*^−/−^ (n = 7) mice. (**G**) Representative images of colon tumors in 120-day-old littermate *Apc*^Min/+^ and *Apc*^Min/+^*Irf9*^−/−^ mice. (**H**) Number of colon tumors in 120-day-old *Apc*^Min/+^ (n = 5) and *Apc*^Min/+^*Irf9*^−/−^ mice (n = 5) mice. (**I**) Percentage of tumors of various sizes in 120-day-old littermate *Apc*^Min/+^ (n = 5) and *Apc*^Min/+^*Irf9*^−/−^ (n = 5) mice. Each symbol represents 1 individual mouse (**C**,**F**,**H**). * *p* < 0.05; ** *p* < 0.01; **** *p* < 0.0001. Two-tailed *t*-test (**C**,**F**,**H**) and one-way ANOVA (**A**) were used. Data are represented as mean ± SEM in (**A**,**C**,**F**,**H**).

IFN signaling is a critical branch of the host immune system that modulates tumorigenesis [33]. Within the type I IFN signaling pathway, STAT1, STAT2, and IRF9 make up a central signaling complex ISGF3, which modulates the host immune response by regulating the production of cytokines, chemokines, and ISGs. The function of ISGF3 in CRC, however, is unclear, as its constituents have varying roles. STAT1 restricts gut inflammation and suppresses CRC via modulating cellular proliferation, inflammation, and apoptosis [25]. However, STAT2 is detrimental in CRC and leads to increased expression and secretion of proinflammatory cytokines in the colon [24]. The role of the third component of ISGF3, IRF9, is unknown in CRC. Therefore, to investigate the role of IRF9 in colorectal tumorigenesis, we subjected cohorts of wild type (WT) and *Irf9*^−/−^ mice to a single injection of the DNA-damaging agent AOM and administered three cycles of DSS in the drinking water to induce the standard murine CRC model, as described previously [21]. We then monitored body weight changes and tumor prevalence in WT and *Irf9*^−/−^ littermate mice 80 days after AOM injection. *Irf9*^−/−^ mice lost less body weight compared with WT mice after DSS cycles (Figure 1A). The colons of *Irf9*^−/−^ mice had a reduced tumor burden in terms of both number of tumors and size compared with WT mice (Figure 1B–D). Histopathological analysis showed that the colons of *Irf9*^−/−^ mice had reduced thickening compared with WT mice (Figure 1E,F). Histological hallmarks associated with inflammation, ulceration, hyperplasia, and the extent or severity of damage were also reduced in the colon of *Irf9*^−/−^ mice compared with the corresponding littermate WT mice (Figure 1F). Together, these findings suggest that IRF9 plays a role in promoting tumorigenesis in CRC.

To further confirm the tumor-promoting role of IRF9 in CRC, we used a spontaneous mouse model of colon cancer. In this model, mice containing a heterozygous mutation in the gene encoding adenomatous polyposis coli (*Apc*^Min/+^) were crossed with *Irf9*^−/−^ mice to generate *Apc*^Min/+^*Irf9*^−/−^ littermate mice. We found that *Apc*^Min/+^*Irf9*^−/−^ mice had a reduced tumor burden compared with *Apc*^Min/+^ control mice in terms of both the number and size of tumors (Figure 1G–I). Overall, these data suggest that IRF9 is detrimental in the development of colorectal tumorigenesis.

### 3.2. IRF9 Promotes DSS-Induced Colonic Inflammation

**Figure 2:**
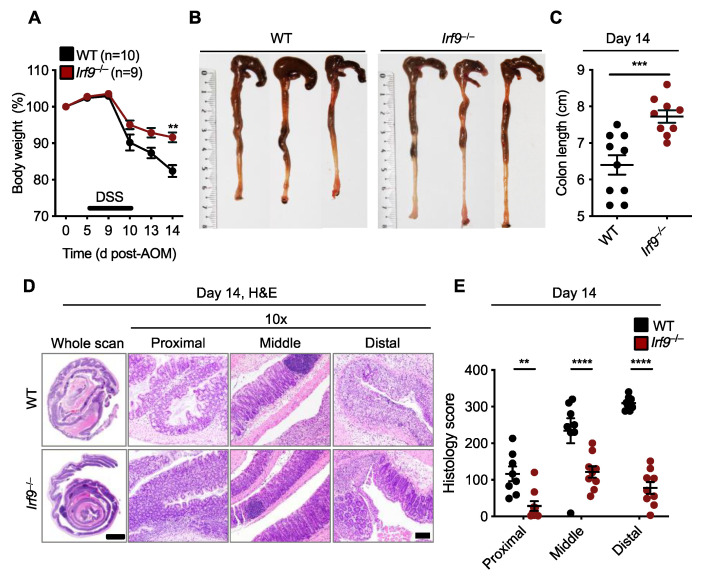
**IRF9-deficient mice are resistant to DSS-induced colonic inflammation.** (**A**) Body weight change of wild type (WT) (n = 10) and *Irf9*^−/−^ (n = 9) littermate mice. (**B**,**C**) Representative images of colon (**B**) and length of colon (**C**) in WT (n = 10) and *Irf9*^−/−^ (n = 9) mice 14 days after azoxymethane (AOM) injection. (**D**) Representative hematoxylin and eosin (H&E) staining of colon; scale bar, 2 mm for whole scan and 500 μm for proximal, distal, and middle colon. (**E**) Histological scores 14 days after AOM injection. Each symbol represents an individual mouse (**C**,**E**). ** *p* < 0.01; *** *p* < 0.001; **** *p* < 0.0001. One-way ANOVA (**A**); two-tailed *t*-test (**C**); and two-way ANOVA (**E**) were used. ns, not statistically significant. Data are from one experiment representative of three independent experiments (**A**–**E**).

Inflammation can drive the development of colitis-associated CRC [18]. Therefore, we investigated whether the reduced tumor development in the colons of *Irf9*^−/−^ mice could be due to reduced colonic inflammation. We subjected cohorts of WT and *Irf9*^−/−^ littermate mice to an AOM injection followed by a single round of DSS to induce colitis. Body weight change and inflammatory responses were monitored in these mice for 14 days after AOM injection. *Irf9*^−/−^ mice lost less body weight and had reduced shortening of the colon after the single round of DSS treatment (Figure 2A–C). Hematoxylin and eosin staining showed reduced cellular infiltration and less damage of the colon in *Irf9*^−/−^ mice on day 14 compared with WT mice (Figure 2D). Moreover, all the histologic parameters assessed, namely inflammation, ulceration, and hyperplasia, and the extent or severity of damage, were significantly reduced in proximal, middle, and distal regions of the colons of *Irf9*^−/−^ mice (Figure 2D,E). Overall, these data suggest that IRF9 promotes DSS-induced colitis.

In addition to inflammation, dysregulation of or acquired resistance to cell death can regulate tumorigenesis. The IFN signaling pathway is known to be important in this process; for example, IRF1 acts as a tumor suppressor in a mouse model of CRC and in human cancer cells by promoting PANoptosis [21,22]. PANoptosis is an innate immune inflammatory programmed cell death pathway dependent on PANoptosomes, caspase(s)-containing complexes with or without inflammasome components and RHIM-containing proteins [15,21,22,34,35,36,37,38,39,40,41,42,43,44,45,46,47,48,49]. Given that *Irf9*^−/−^ mice develop fewer CRC tumors, we hypothesized that cell death pathways may be modulated in the colon of these mice. However, we found no consistent differences in the activation of cell death components (gasdermin D and gasdermin E, CASP3, CASP7, and CASP8) at day 14 in the colons of AOM/DSS-treated WT and *Irf9*^−/−^ mice (Supplemental Figure S1), suggesting that reduced tumor burden in *Irf9*^−/−^ mice is due to the role of IRF9 in colitis and not in colonic cell death regulation in response to AOM/DSS.

### 3.3. IRF9 Is Not Required for Inflammatory Signaling in the Colon but Impacts IL-6 Production

**Figure 3:**
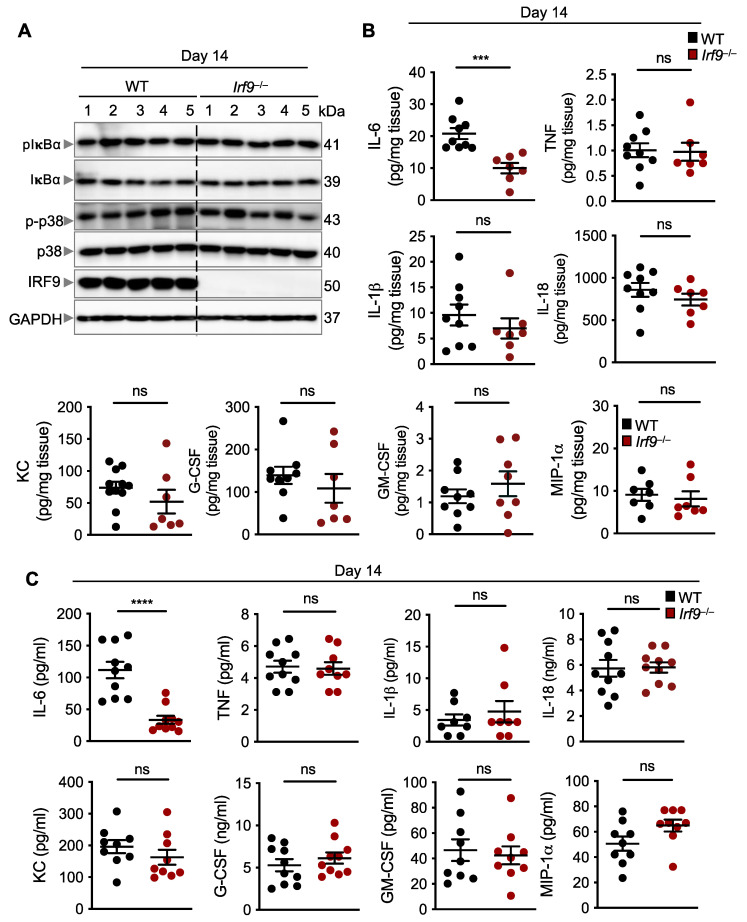
**IRF9 does not regulate inflammatory signaling but does regulate IL-6 production in the colon.** (**A**) Immunoblot analysis of phosphorylated and total IκBα (pIκBα and IκBα, respectively), phosphorylated and total p38 (p-p38 and p38, respectively), IRF9, and GAPDH (loading control) in colon lysates from wild type (WT) and *Irf9*^−/−^ mice. Each lane corresponds to an individual mouse. (**B**) Levels of inflammatory cytokines in the colon of WT (n = 7–9) and *Irf9*^−/−^ (n = 7–8) mice 14 days after AOM injection. (**C**) Levels of inflammatory cytokines in the serum of WT (n = 8–10) and *Irf9*^−/−^ (n = 8–10) mice 14 days after AOM injection. Each symbol represents an individual mouse in (**B**,**C**). ns, not statistically significant. *** *p* < 0.001, **** *p* < 0.0001. Two-tailed *t*-test was used in (**B**,**C**). Data are represented as mean ± SEM in (**B**,**C**).

Inflammation is one of the major hallmarks of cancer, and inflammatory signaling can modulate the transcription and translation of several pro- and anti-inflammatory cytokines that play a role in tumorigenesis [50]. To investigate whether inflammatory signaling pathways were altered in IRF9-deficient mice after DSS administration, we measured the activation of NF-κB and p38 mitogen activated protein kinase (MAPK) and found them to be similar in the colon of WT and IRF9-deficient mice 14 days after AOM/DSS treatment (Figure 3A). We also evaluated the production of inflammatory cytokines, which can affect tumor development by modulating several signaling pathways in paracrine and autocrine manners. We found that the production of TNF, IL-1β, IL-18, KC, G-CSF, GM-CSF, and MIP-1α was similar in both the colons and serum of WT and IRF9-deficient mice (Figure 3B,C). In contrast, the production of IL-6 was significantly lower in the colon and serum of IRF9-deficient mice compared with WT mice (Figure 3B,C). Overall, these data suggest that while IRF9 is not required for mediating inflammatory signaling in the colon, it does influence the production of IL-6.

### 3.4. IRF9 Transcriptionally Regulates the Production of IL-6

**Figure 4:**
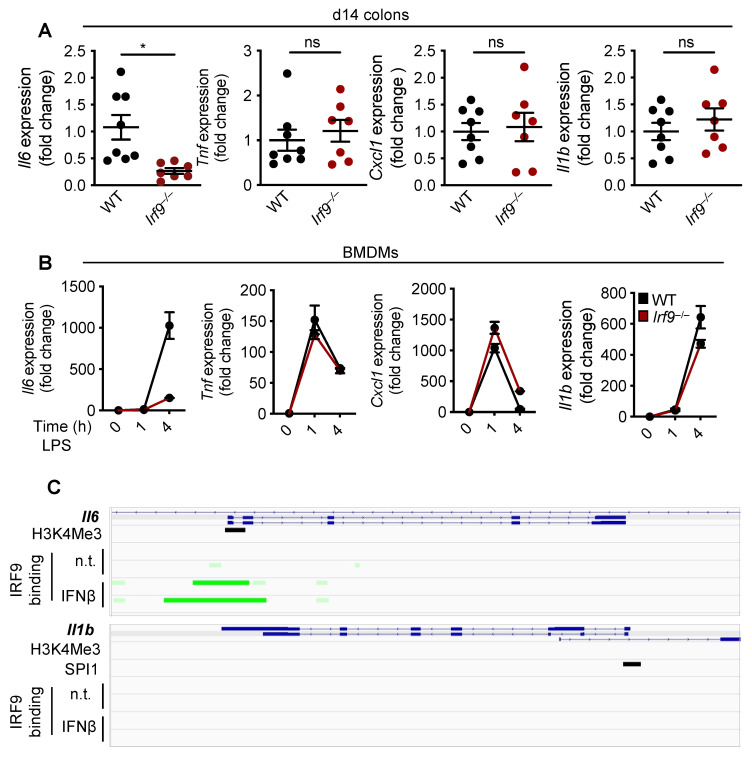
**IRF9 regulates IL-6 transcription.** (**A**) Transcript expression of *Il6*, *Tnf*, *Cxcl1* (*Kc*), and *Il1b* in the colon tissues of wild type (WT) (n = 8) and *Irf9*^−/−^ (n = 7) mice14 days after AOM injection and (**B**) in LPS-stimulated WT and *Irf9*^−/−^ bone marrow-derived macrophages (BMDMs) at 1 and 4 h post-stimulation. (**C**) ChIP-Seq analysis of IRF9 binding to the *Il6* (top) and *Il1b* (bottom) promoter. Green highlights the IRF9 binding site. Each symbol represents an individual mouse in **A**. ns, not statistically significant. * *p* < 0.05. Two-way ANOVA was used in **A**. Data are represented as mean ± SEM in (**A**,**B**).

In primary tumors from patients with CRC, reduced IL-6 expression is directly associated with improved disease-free survival rates [51]. Several ligands including LPS, TNF, and IL-1α upregulate the expression of IL-6 [52]; additionally, various transcription factors, including NF-κB, AP1, and C/EBP, upregulate the gene expression of *Il6* by binding to its promoter region [52]. To determine whether IRF9 also regulates *Il6* transcription, we first measured the mRNA expression of *Il6* in the colons of WT and IRF9-deficient mice. We found that the expression of the *Il6* transcript was significantly reduced in the colons of IRF9-deficient mice 14 days after AOM/DSS treatment compared with expression in WT mice (Figure 4A). To understand the specificity of IRF9 for *Il6* transcription, we measured the mRNA expression level of other inflammatory cytokines in the colon. We found that the mRNA expression of *Tnf*, *Cxcl1 (Kc)*, and *Il1b* was similar in the colons of both the WT and IRF9-deficient mice (Figure 4A), suggesting that IRF9 specifically regulates *Il6* expression in the colon. Additionally, we stimulated bone marrow-derived macrophages (BMDMs) from WT and IRF9-deficient mice with LPS, a known inducer of pro-inflammatory cytokine expression, and measured *Il6*, *Tnf*, *Cxcl1*, and *Il1b* gene expression and observed reduced expression of *Il6* in IRF9-deficient BMDMs (Figure 4B). However, gene expression of other inflammatory cytokines including *Tnf*, *Cxcl1*, and *Il1b*, was similar in both the WT and IRF9-deficient macrophages stimulated with LPS.

To further understand how IRF9 upregulates *Il6* transcription, we analyzed publicly available ChIP-Seq datasets for IRF9 transcription factor binding sites. We identified an enrichment of IRF9 binding to the promoter region of *Il6*. However, there was no enrichment of IRF9 binding to the promoter of other inflammatory cytokines such as *Il1b* (Figure 4C), suggesting that IRF9 may specifically bind to the *Il6* promoter to regulate its transcription. Overall, these data suggest that IRF9 transcriptionally regulates *Il6* gene expression.

### 3.5. IRF9-Mediated IL-6 Production Promotes STAT3 Activation

**Figure 5:**
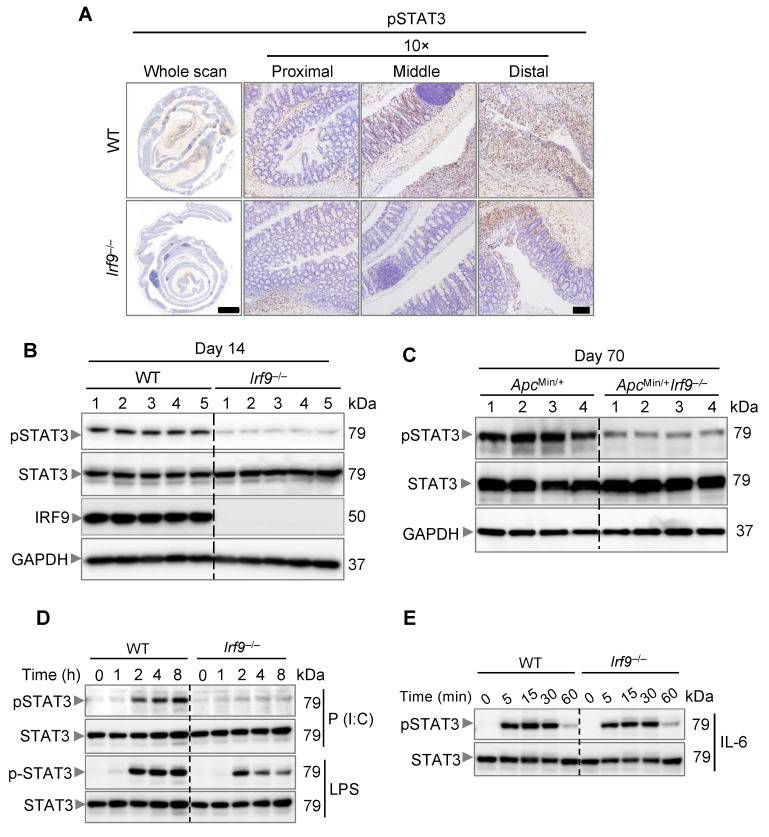
**IRF9 drives STAT3 activation.** (**A**) Representative images of pSTAT3 staining of colon tissues from AOM/DSS-treated wild type (WT) and *Irf9*^−/−^ mice 14 days after AOM injection. Tissues were counterstained with hematoxylin. (**B**) Immunoblot analysis of phosphorylated and total STAT3 (pSTAT3 and STAT3, respectively) and IRF9 in the colon of WT and *Irf9*^−/−^ mice 14 days after AOM injection. Each lane corresponds to an individual mouse. (**C**) Immunoblot analysis of pSTAT3 and STAT3 in the colon of *Apc*^Min/+^ and *Apc*^Min/+^*Irf9*^−/−^ mice at day 70. Each lane corresponds to an individual mouse. (**D**) Immunoblot analysis of pSTAT3 and STAT3 in WT and *Irf9*^−/−^ bone marrow-derived macrophages (BMDMs) stimulated with poly(I:C) (P (I:C)) or LPS for the indicated time. (**E**) Immunoblot analysis of pSTAT3 and STAT3 in WT and *Irf9*^−/−^ BMDMs stimulated with IL-6 for the indicated time. Scale bar, 2 mm for whole scan and 500 μm for proximal, distal, and middle colon.

IL-6 is known to lead to the activation of STAT3, which is a proto-oncogene that can induce tumor formation [53]. STAT3 deficiency can reduce the tumor burden in AOM/DSS-treated mice [53,54]. Given that IRF9 transcriptionally regulated *Il6* expression, we next examined whether IRF9-deficient mice had reduced STAT3 activation. We observed that colons of IRF9-deficient mice showed reduced pSTAT3 staining compared with those in WT mice 14 days after AOM/DSS treatment (Figure 5A). We also performed immunoblotting and found reduced pSTAT3 in the colon lysates from IRF9-deficient mice compared with that in WT mice (Figure 5B). Additionally, in the spontaneous colon cancer model, we observed reduced pSTAT3 in *Apc*^Min/+^*Irf9*^−/−^ mice compared with their *Apc*^Min/+^ littermates at day 70 (Figure 5C). To further link IRF9 to IL-6 and STAT3 signaling, we stimulated WT and IRF9-deficient BMDMs with LPS or poly(I:C), which induce IL-6 production. We found that IRF9-deficient BMDMs showed reduced STAT3 activation compared with WT BMDMs in response to both LPS and poly(I:C) (Figure 5D). Combined with our finding that IRF9 deficiency reduced IL-6 transcription (Figure 4), these data suggest that STAT3 activation required IRF9-mediated IL-6 upregulation. Furthermore, when we stimulated WT and IRF9-deficient BMDMs with IL-6, we found similar activation of STAT3 in both (Figure 5E), showing that it is IL-6 production and not the ability of IL-6 to activate STAT3 that is inhibited in IRF9-deficient cells. Overall, these findings suggest that IRF9-mediated IL-6 upregulation and production is required for STAT3 activation, linking IRF9 to the IL-6/STAT3 signaling axis.

The role of IL-6 and STAT3 in promoting colorectal cancer has previously been reported [53]. The tumor-promoting effect of IL-6 largely depends upon STAT3 activation; IL-6-deficient mice have reduced tumor growth and have abrogated STAT3 activation, suggesting the importance of IL-6 and STAT3 signaling in promoting colorectal tumorigenesis. Additionally, STAT3 deletion reduces AOM/DSS-induced colorectal tumorigenesis [53]. Overall, these previously published findings establish the role of the IL-6-STAT3 signaling axis in colorectal tumorigenesis. Combined with our in vivo and in vitro results showing that IRF9 mediates IL-6 production for STAT3 activation, these data suggest that IRF9 drives the activation of STAT3 in the colon to promote colorectal tumorigenesis.

## 4. Discussion and Conclusions

IFN signaling has an important role in modulating tumorigenesis [12]. Although the general role of IFN signaling in CRC has been studied by several groups, the functions of the individual proteins in the IFN signaling pathway are distinct in modulating CRC [23,24,55], and the specific functions of IRF9 have remained unknown. Herein, we discovered that IRF9-deficient mice were resistant to the development of tumors in both the AOM/DSS-induced mouse model of CRC and in a spontaneous CRC tumor model. While IRF9 was not required to produce many of the major inflammatory cytokines or for the activation of NF-κB and p38 MAPK, it was required for the production of IL-6, via transcriptional regulation, and the subsequent activation of STAT3.

Diverse functions for IRF9 have been described in different tumor cells. IRF9 facilitates the anti-proliferative effects of IFN-α in prostate cancer cells [56], reduces tumor growth in renal carcinoma cells [57], and represses cell growth in acute myeloid leukemia [58]. In contrast, IRF9 can also have oncogenic properties [59,60]. Oncogenes can be associated with cell death pathways [61], and in vitro, IRF9 has been previously shown to modulate cell death [62,63]. However, we did not observe any consistent differences in the activation of cell death effectors in the colons of WT and IRF9-deficient mice, suggesting that IRF9 does not modulate cell death to promote colorectal tumorigenesis in vivo. This is likely due to inherent differences between the in vitro system, where primary cell lines were stimulated with a specific ligand to activate a particular cellular signaling pathway, and the in vivo system, where multiple ligands will be present simultaneously to modulate several cellular signaling pathways.

We observed that rather than impacting cell death pathways, IRF9 modulated the production of IL-6. Levels of IL-6 are known to be higher in the serum of *Apc*^Min/+^ mice, and, in the cachexia model, the intestinal tumor development in these mice can be rescued by IL-6 depletion [64]. Additionally, crossing *Apc*^Min/+^ mice with *Il6*^−/−^ mice significantly reduces the overall intestinal polyp number, providing further evidence for a pathogenic role of IL-6 in colorectal tumorigenesis [64]. We hypothesize that IL-6 production and STAT3 activation would also be significantly reduced in *Apc*^Min/+^*Irf9*^−/−^ mice, and future studies will be required to understand how IRF9 promotes tumor formation in *Apc*^Min/+^ mice specifically.

IL-6 is typically produced by immune cells in response to sensing pathogen- and damage-associated molecular patterns, and it can act on intestinal epithelial cells in a paracrine manner to promote their proliferation [65]. Here, IRF9-deficient BMDMs had reduced *Il6* transcript expression in response to LPS. Similarly, AOM/DSS-treated IRF9-deficient mice produced less IL-6 in the colon. Therefore, IL-6 could be produced by the cells of the hematopoietic compartment in vivo, allowing IL-6 to act on the epithelial cells and drive STAT3 activation. Nevertheless, future studies are required to understand the source of IL-6 production during colitis and the in vivo mechanism of STAT3 activation.

Overall, our study found a non-canonical function of IRF9 in the activation of the IL-6/STAT3 signaling axis to promote the development of CRC. These findings highlight the potential impact of targeting IL-6/STAT3 to modulate CRC, which could help inform future strategies for the treatment of CRC and other IRF9- and IL-6-mediated pathologies.

## Data Availability

ChIP-Seq data can be accessed in GEO under accession numbers GSE115435 for IRF9 and GSM1441327 for H3K4Me3. All other datasets generated or analyzed during this study are included in the published article.

## Supplementary Materials

The following supporting information can be downloaded at: https://www.mdpi.com/article/10.3390/cancers14040919/s1, Figure S1: IRF9 does not regulate cell death in colon; Table S1: Real-time qPCR primer sequences.
