# Supplementary material for: The Transcription Factor IRF9 Promotes Colorectal Cancer via Modulating the IL-6/STAT3 Signaling Axis
Source: Cancers (Basel). 2022 Feb 12;14(4):919. doi: 10.3390/cancers14040919 (PMC8869918; doi:10.3390/cancers14040919)
Supplement: Supplementary file 1 [file cancers-14-00919-s001.zip › cancers-1525940-supplementary.pdf]

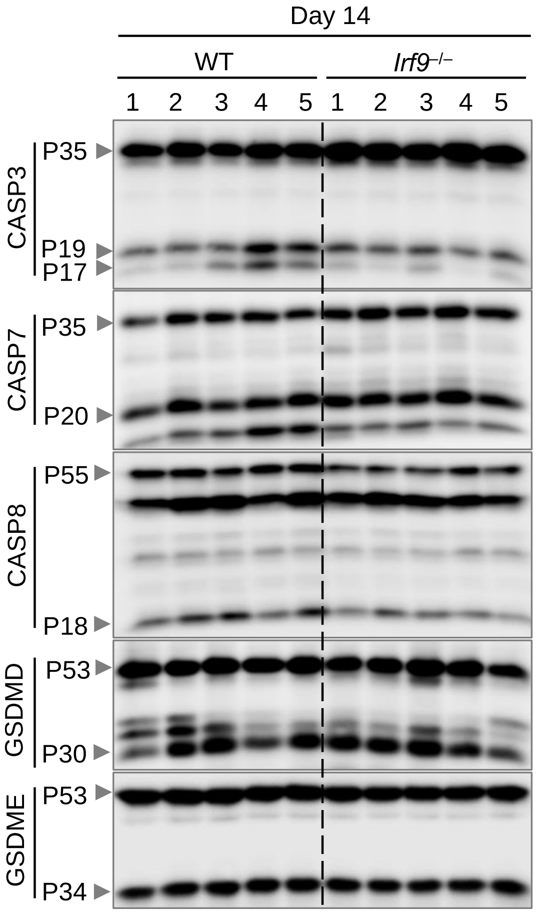

Supplementary Figure S1. IRF9 does not regulate cell death in colon

Immunoblot analysis of caspase-3 (CASP3), caspase-7 (CASP7), caspase-8 (CASP8), gasdermin D (GSDMD), and gasdermin E (GSDME) in the colons of WT and *Irf9*$^{-/-}$ mice 14 days after AOM injection. Each lane corresponds to an individual mouse.

SUPPLEMENTARY TABLE

Supplementary Table S1. Real-time qPCR primer sequences

Target	Primer sequence
mIl6	Forward: 5’-CGTCCCGTAGACAAAATGGT-3’<br>Reverse: 5’-TTGATGGCAACAATCTCCAC-3’
mCxcl1	Forward: 5’- CAATGAGCTGCGCTGTCAGTG-3’<br>Reverse: 5’-CTTGGGGACACCTTTTAGCATC-3’
mTnf	Forward: 5’-CATCTTCTCAAAATTCGAGTGACAA-3’<br>Reverse: 5’-TGGGAGTAGACAAGGTACAACCC-3’
mIl1b	Forward: 5’-GATCCACACTCTCCAGCTGCA-3’<br>Reverse: 5’-CAACCAACAAGTGATATTCTCCATG-3’
mHprt	Forward: 5’-CTCATGGACTGATTATGGACAGGAC 3’<br>Reverse: 5’-GCAGGTCAGCAAAGAACTTATAGCC 3’
